# Estimating functions for visual field progression in newly diagnosed exfoliation glaucoma patients in Sweden

**DOI:** 10.1038/s41598-023-48336-6

**Published:** 2023-11-28

**Authors:** Marcelo Ayala

**Affiliations:** Eye Department, Skaraborg Hospital, Skövde, Sahlgrenska Academy, Gothenburg University & Karolinska Institute, 541 85 Skövde, Sweden

**Keywords:** Eye diseases, Ocular hypertension, Glaucoma

## Abstract

This study aimed to determine whether glaucoma progression was linear or not in newly diagnosed exfoliation glaucoma patients. A total of 96 patients with newly diagnosed exfoliation glaucoma were included. These patients were required to undergo at least seven visual field tests within 3 years (± 1 month), and all were treated at the time of inclusion. The study was a non-randomized, prospective cohort study. The outcome of the study was visual field progression. Progression was assessed based on mean deviation (MD), visual field index (VFI), and “Guided Progression Analysis”. The MD and VFI values were plotted against time, and distribution and curve fit were calculated. The results showed that the general rate of progression of the cohort was − 3.84 (± 2.61) dB for the MD values and 9.66 (± 6.25)% for the VFI values over 3 years. The best-fitted curve for MD and VFI values in the 36 months period was significant for both linear and exponential curves (*p* ≤ 0.001; *p* ≤ 0.001). However, in the MD group, the F and the R^2^ values were higher for exponential than for linear function (linear: F = 42.60, R^2^ = 0.059; exponential: F = 53.26, R^2^ = 0.073). The opposite results were found among VFI values. The F and the R^2^ values were slightly better for linear than for exponential (linear: F = 37.22, R^2^ = 0.052; exponential: F = 35.55, R^2^ = 0.050). In conclusion, the study found that visual field progression between diagnosis and 18 months seemed to be exponential. However, after 18 months, the IOP reduction effects probably ameliorated progression, making the curve linear.

## Introduction

Mathematical models have been widely utilized in the field of science. These models are based on empirical testing and theory, attempting to simplify and describe complex processes using mathematical equations. Moreover, these models can predict outcomes under different conditions. In recent years, mathematical models have been on the rise in health sciences^1^. The use of artificial intelligence (AI) in health sciences poses challenges, including developing accurate mathematical models to predict results.

Glaucoma is a prevalent eye condition that can result in the loss of ganglion cells in the retina, leading to a decline in the visual field. In Sweden, primary open-angle (POAG) and exfoliation glaucoma (EXFG) are this disease’s most frequent clinical presentations^2^. The EXFG is caused by the accumulation of protein material in the eye’s trabecular meshwork, thus increasing the intraocular pressure (IOP). Although the cause and mechanisms of exfoliation are unclear, several genes have been linked to the disease^3,4^. Previous studies have suggested that EXFG is a rapidly progressive form of glaucoma^5–7^, and visual field testing is still considered the gold standard for assessing glaucoma progression, as the European Glaucoma Society recommends^8^. According to the Swedish guidelines for glaucoma care, at least seven visual fields (one baseline and six follow-up) are required over 3 years to detect progression^9^. As visual fields vary among individuals, several tests are needed to conclude. Visual field analysis is the preferred method for estimating glaucoma progression. Other methods, like anatomical measurements of the optic nerve, are in use, but their relevance for determining glaucoma progression is still under discussion^8^.

Mathematical models have long been utilized in medicine to explain various phenomena. In medicine, regression analyses are commonly employed to investigate the relationship between a predictor (cause) and an outcome (effect). These analyses are based on different mathematical models depending on the distribution of the data. In linear regression, the outcome increases linearly with the predictor, while in exponential regression, the outcome (glaucoma progression, for example) does not necessarily change directly with the predictor (time). A linear function always has a straight-line graph, whereas an exponential function yields a curved graph. Previous studies on glaucoma progression have suggested that glaucoma damage progresses linearly, but the evidence supporting this is limited^10–12^.

The present study aimed to determine whether visual field deterioration in newly diagnosed EXFG patients developed linearly or not.

## Materials and methods

This was a 6 years non-randomized prospective cohort study that included all patients newly diagnosed with EXFG. The study recruited patients from January 1, 2012 to December 31, 2017, who attended the Ophthalmology Department at Skaraborg Hospital. Patients with a newly diagnosed EXFG were asked to participate in the study. All patients were provided oral and written information about the study. All patients signed the informed consent form. The Regional Ethical Review Board in Gothenburg, Sweden, granted ethical approval for the study (DN:119-12). All experiments were performed in accordance with relevant guidelines and regulations. The study followed the tenets of the Helsinki Declaration.

Patients newly diagnosed with EXFG, according to the European Glaucoma Society (EGS) guidelines, and aged 85 years or less were included. Patients who were unable to perform reliable visual field tests, had advanced visual field damage, suffered from other significant eye diseases, or could not be followed up for 3 years due to dementia, general illness, or moving to another part of the country were excluded. Patients who underwent glaucoma surgery, uncomplicated cataract surgery or SLT were not excluded from the study.

During the recruitment visit, all patients received ophthalmological exams. Their age and sex were also noted. Patients with unilateral or bilateral glaucoma were observed, and when bilateral glaucoma, one eye was randomly selected for the study.

Visual acuity was evaluated using a Snellen chart, and IOP was measured with a Goldmann’s applanation tonometer. The average of three IOP measurements was taken. Central corneal thickness (CCT) was measured with an ultrasound device (Tomey Pachymetry; Tomey Corp., Nagoya 451–0051, Japan). A gonioscopy was performed with a goniolens with undilated pupils to inspect the trabecular meshwork.

All patients were tested using a Humphrey Field Analyzer (Carl-Zeiss, Straße 22, 73447 Oberkochen, Germany) to perform a visual field test with the 24-2 technique of the Swedish Interactive Threshold Algorithm (SITA fast). The 24-2 SITA fast is the commonest technique for glaucoma evaluation used in Sweden. The patient’s pupils were then dilated, and exfoliation was observed in the anterior region of the eye with a slit lamp. A 90-D lens was used to examine the optic nerve, and the cup-to-disc (C/D) ratio was recorded.

All patients were treated with IOP-lowering eye drops at inclusion. The IOP was measured one month after the recruitment appointment to evaluate the medication’s effect. Over the following 3 years (± 1 month), patients underwent examinations every six months (± 1 month). Visual acuity, IOP, and visual fields were assessed at each visit. The patients completed at least seven visual fields in total, including one at the beginning (baseline) and six throughout the 3 years follow-up. All patients followed the Swedish Guidelines for glaucoma care^9^. If the IOP needed to be lowered, new drugs were given and/or SLT was performed.

### Endpoints and functions definitions

The study’s primary aim was to examine the progression of visual field loss in patients with newly diagnosed EXFG. To measure this, three methods were employed—two continuous variables and one binomial variable (Yes/No).

The first method utilized mean deviation (MD) values, an older technique to measure visual fields that can be altered if the patient has cataracts.

The second method used visual field index (VFI) values, calculated as a percentage of a normal visual field’s 100% VFI.

The third technique, GPA, was an automated process executed by the device that compared every point with similar points found in earlier assessments and classified progression as no, possible, or likely. Unlike MD and VFI, which are “trend analyses”, GPA is an “event analysis”. For analytical purposes, the results of GPA were divided into progression and no progression (binary variables), where progression includes both possible and likely progression. In Sweden, GPA and VFI are the most used tests in clinical practice to estimate glaucoma visual field deterioration.

In the present study, the linear function had the form ${\text{y}} \, \text{=} \, {\text{mx}} \, \text{+} \, {\text{b}}$, (1) where “y” was the glaucoma progression (MD or VFI), “x” was time, “m” was the coefficient of the independent variable (also known as “slope”) and “b” was the constant term. In the case of an exponential function, the form was $y = b*e^{m*x}$ (2) where “y” was the glaucoma progression (MD or VFI), “b” was the y-intercept, “e” was the Euler’s number, “m” was the slope, and “x” was the independent variable (time).

### Statistics

We utilized IBM’s SPSS software (Armonk, NY 10504, USA) to conduct statistical analyses. Initially, we used a T-test or Chi-square test to test the baseline clinical characteristics of the cohort to identify any differences based on GPA analysis. We then employed the Kolmogorov–Smirnov test in SPSS to investigate the distribution of MD and VFI values. After testing for normality, the MD and VFI values at different time points were analyzed using analysis of variance (ANOVA) and subsequent Tukey’s honest significant difference (HSD). Then, we plotted all values on an Excel graph and calculated the best-fit curve using SPSS, where the MD values were converted to positive values for curve estimation. For instance, a value of − 10 dB was transformed to 10 dB.

The analysis of the function curves was done in two stages. Firstly, all the MD and VFI values for 36 months were tested using SPSS. The functions chosen at this first were linear, exponential, logarithmic, quadratic, and cubic. Only significant functions were taken to the next stage. If two or more functions showed significance, only the two with the highest R^2^ and F-value were considered for the next stage^13^.

The second stage was to analyze the values divided into groups based on time (0–18 and 18–36) and according to the GPA analysis. Only the two functions with the highest R^2^ and F-value from the first step were used in this second stage. The significance level for all the calculations was set at *p* ≤ 0.05.

## Results

In this study, 96 patients were originally included. The patients included in this study had an average age of 70.33 (± 6.04) years, with an almost equal distribution of 49 females and 47 males (51/49%). The mean visual acuity in Snellen units was 0.8 (± 0.23), and the mean IOP at inclusion was 32.52 (± 5.54) mmHg. Among the patients with exfoliation glaucoma, 66 had it in one eye only, while 30 had it in both eyes (69/31%). The mean CCT was 546.06 (± 34.08) µm. The average cup/disc ratio was 0.79 (± 0.09). Only 15 out of 96 patients (15%) had undergone cataract surgery (pseudophakia) at the time of inclusion.

All patients were monitored for 3 years during the study, with a margin of plus or minus one month. The entire group had a decrease in MD of − 3.84 (± 2.61) dB over 3 years. This translates to an average yearly visual field deterioration of − 1.28 dB or 4.2% per year. The rate of progression for the visual field index (VFI) was 9.66 (± 6.25) % over 3 years, which equates to approximately 3.22% per year. In relative terms, this means a visual field deterioration of 3.2% per year (since VFI is already a percentage value).

At the end of the study, the patients underwent a GPA evaluation, which divided the group into two categories: those who showed progression and those who did not. Altogether, 37 patients did not experience any visual field deterioration, while 59 patients showed disease progression. At the beginning of the study, there was a significant difference in intraocular pressure (IOP) values between the two groups. Patients who progressed had an average IOP of three mmHg higher than those who did not progress (t-test; *p* = 0.005). Age was also a significant factor, with progressing patients being an average of 3 years older than non-progressing patients (t-test; *p* = 0.03). The MD and VFI values at the beginning of the study were significantly different between the two groups. MD values were twice as high for progressors than non-progressors (t-test; *p* < 0.001). Conversely, VFI values at the beginning of the study were approximately 10% lower for progressors than non-progressors (t-test; *p* < 0.001). Over a span of 3 years, cataract surgery was performed on patients from both the no-progress and progress groups, with six patients in the former and eight in the latter. However, there was no difference between the two groups, as confirmed by a Chi-square test (*p* = 0.54). On the opposite, a significantly higher number of patients^20^ in the progress group received SLT treatment compared to the no-progress group^2^ (Chi-square; *p* ≤ 0.001). Furthermore, the progress group had higher usage of medications (around three) than the no-progress group (around two) (t-test; *p* ≤ 0.001). The rate of progression was also considerably higher in the progress group in contrast to the no-progress group. This was evident from the MD values, which showed that progressors had double the rate of progression compared to non-progressors (− 1.38 dB/year vs. − 0.6 dB/year) (t-test; *p* ≤ 0.001). Comparable results were obtained when calculating the rate of progression using VFI values, with progressors exhibiting a rate around three times higher than no progressors (2.95%/year vs. 0.93%/year) (t-test; *p* ≤ 0.001). Table 1 provides additional information on these findings.

**Table 1:** General clinical characteristics of the patients at inclusion and at 36 months based on the GPA analysis.

	No progress (N = 37)	Progress (N = 59)	Test	*P* value
Age at inclusion (years) (SD)	71.29 (± 6.61)	74.15 (5.59)	T-test	0.03*
Sex (F/M) (%)	21/16 (57/43)	29/30 (49/51)	Chi-square	0.40
IOP at inclusion (mmHg) (SD)	30.54 (5.88)	33.75 (5.01)	T-test	0.005*
IOP at 36 months	17.01 (1.85)	17.98 (1.78)	T-test	0.81
MD at inclusion (dB) (SD)	− 3.7 (3.44)	− 7.58 (4.96)	T-test	< 0.001*
MD at 36 months (dB) (SD)	− 5.41 (3.54)	− 12.42 (5.81)	T-test	< 0.001*
VFI at inclusion (%) (SD)	92.05 (9.80)	82.61 (14.72)	T-test	< 0.001*
VFI at 36 months (%) (SD)	88.54 (10.09)	72.76 (17.25)	T-test	< 0.001*
Cataract surgery under follow-up (%)	6/31 (16/84)	8/51 (14/86)	Chi-square	0.54
SLT treatment under follow-up (%)	2/35 (5/95)	20/39 (34/66)	Chi-square	< 0.001*
Number of medicines at 36 months (SD)	2.10 (0.81)	3.06 (0.73)	T-test	< 0.001*

*GPA* guided progression analysis, *SD* standard deviation, *F* female, *M* male, *VA* visual acuity, *CCT* central corneal thickness, *IOP* intraocular pressure, *MD* mean deviation, *VFI* visual field index.

*Significant values at *p* ≤ 0.05.

**Figure 1:**
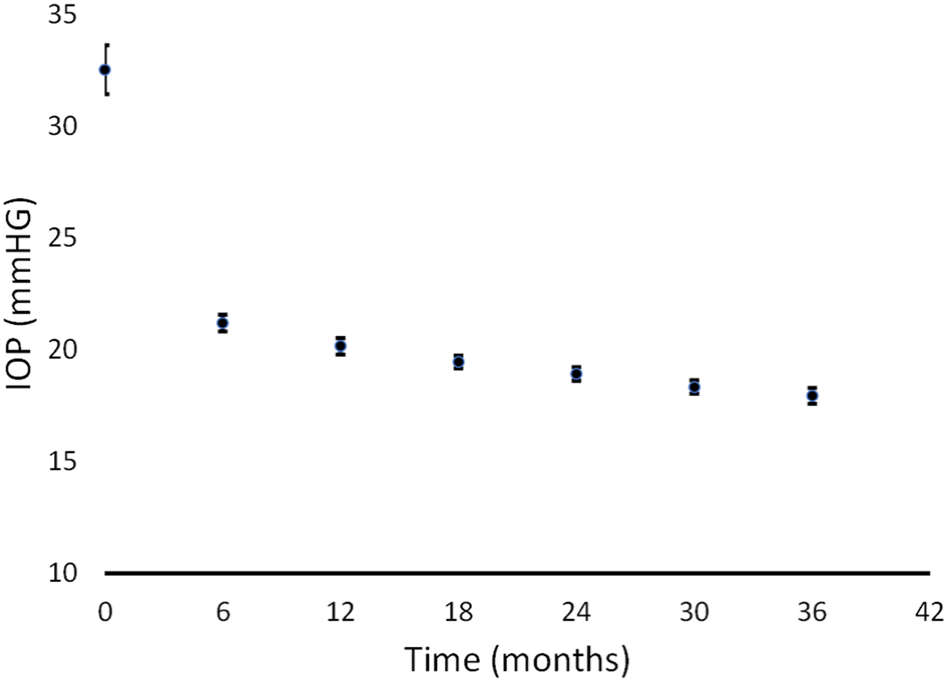
Evolution of the IOP values in the 3 year follow-up period. The bars represent the 95% confidence interval for the mean.

On average, the initial IOP for the entire group was 32.52 mmHg (± 5.54), and at the 6 month check-up, it was 21.19 mmHg (± 1.91). While there was a difference in IOP between those who progressed and those who did not at the beginning of the study, this discrepancy was not present at six months or in the following follow-up periods. Figure 1 illustrates the changes in IOP values.

**Figure 2:**
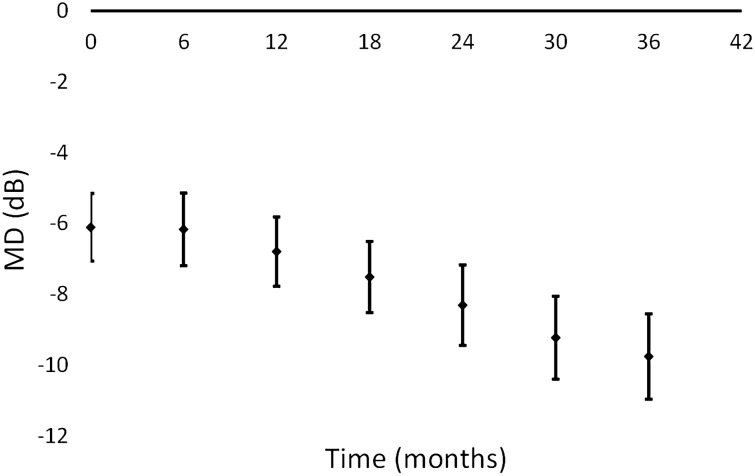
Evolution of the MD values in the 3 year follow-up period. The bars represent the 95% confidence interval for the mean.

**Figure 3:**
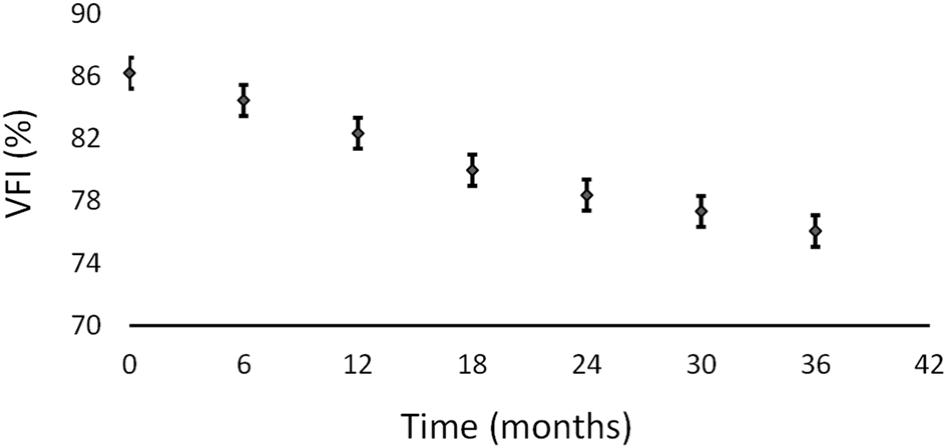
Evolution of the VFI values in the 3 year follow-up period. The bars represent the 95% confidence interval for the mean.

Figures 2 and 3 illustrate the MD and VFI values changes over a 3 years follow-up period.

An analysis (Kolmogorov–Smirnov test) was performed to determine MD and VFI values distribution. An analysis of variance (ANOVA) was performed to detect the differences among the values, and afterwards, a Tukey’s honest significant difference (HSD) test was performed. The ANOVA results were significant (*p* < 0.001 and *p* < 0.001) for the MD and VFI values. Tukey’s test of the MD values showed a significant difference between the baseline and 24, 30, and 36 months (*p* = 0.03, *p* = 0.001, and *p* < 0.001, respectively). Significant differences were also found between 6, 24, 30, and 36 months (*p* = 0.04, *p* = 0.002, *p* < 0.001, respectively). In addition, significant differences were found at 12, 30, and 36 months (*p* = 0.03, *p* = 0.003, respectively). Similar results were obtained when VFI values were considered.

**Figure 4:**
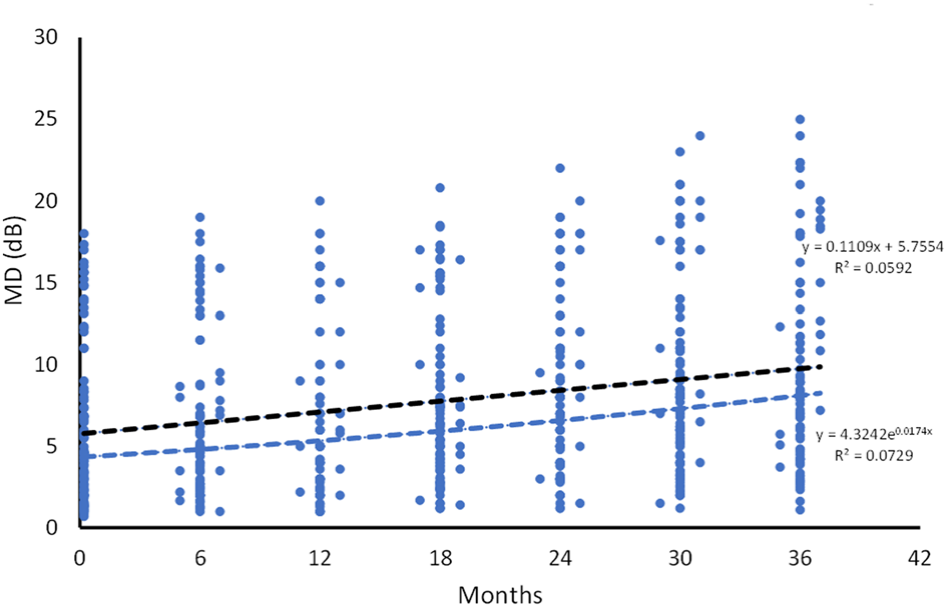
Scatterplot and functions for MD values versus time. The MD values were transformed into positive values from the originals. The black line represents the linear function.

**Figure 5:**
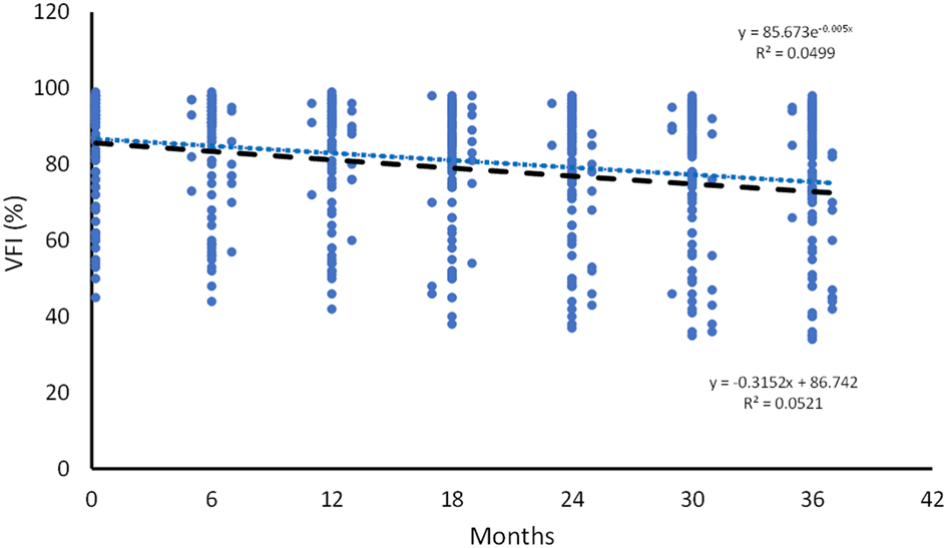
Scatterplot and functions for VFI values versus time. The black line represents the linear function.

The results, obtained using SPSS, indicated that the data followed both normal and exponential distributions, with *p* values of 0.133 and 0.155, respectively. Additionally, Figs. 4 and 5 illustrate the plotted values and curves of MD and VFI.

The most suitable function for MD values was computed using SPSS. For the entire cohort over the 36-month period, the linear and exponential functions were significant (*p* ≤ 0.001/*p* ≤ 0.001). However, the F and R^2^ values were higher for the exponential function than for the linear function (linear: F = 42.60, R^2^ = 0.059; exponential: F = 53.26, R^2^ = 0.073). The data were then divided into two groups and re-analyzed. The first group (0–18 months) demonstrated higher values for the exponential function than the linear function (linear: F = 4.35, R^2^ = 0.011; exponential: F = 7.33, R^2^ = 0.019). However, in the second group (18–36 months), there was almost no difference between the exponential and linear functions (linear: F = 9.90, R^2^ = 0.025; exponential: F = 9.48, R^2^ = 0.024). Please refer to Table 2 for more details.

**Table 2:** Model summary of the MD values for the whole cohort during the 3 years follow-up.

Months	Equation	R^2^	F-value	*P*
0–36	Linear	0.059	42.60	< 0.001*
	Exponential	0.073	53.26	< 0.001*
0–18	Linear	0.011	4.35	0.037*
	Exponential	0.019	7.33	0.007*
18–36	Linear	0.025	9.90	0.002*
	Exponential	0.024	9.48	0.002*

*MD* mean deviation.

*Significant values.

The VFI values were analyzed using a similar approach to the one used for the MD values. Both the linear and exponential curves were statistically significant (*p* ≤ 0.001/*p* ≤ 0.001) for the entire cohort during the 36-month period. The linear function (F = 37.22, R^2^ = 0.052) fit slightly better than the exponential function (F = 35.55, R^2^ = 0.050). The data were then divided into two groups and re-analyzed. The exponential function showed a better fit than the linear function for the 0–18-month period (F = 8.59, R^2^ = 0.022 vs. F = 8.20, R^2^ = 0.020). However, neither function was statistically significant for the 18–36 month period. Refer to Table 3 for more details.

**Table 3:** Model summary of the VFI values for the whole cohort during the 3 years follow-up.

Months	Equation	R^2^	F-value	*P*
0–36	Linear	0.052	37.22	< 0.001*
	Exponential	0.050	35.55	< 0.001*
0–18	Linear	0.020	8.20	0.005*
	Exponential	0.022	8.59	0.004*
18–36	Linear	N.A	N.A	0.07
	Exponential	N.A	N.A	0.06

*VFI* visual field index, *N.A.* non-applicable.

*Significant values.

The cohort was divided into two groups based on their GPA results: no progress and progress. The MD values for the 3 years showed that the function for the no progress group was more exponential than linear (linear: F = 7.85, R^2^ = 0.030; exponential: F = 11.76, R^2^ = 0.051). However, when the results were divided into two subgroups (0–18 and 18–36 months), the difference between these two groups was not significant. On the other hand, the function for progressors was more exponential than linear both in the 0–36 months (linear: F = 47.69, R^2^ = 0.102; exponential: F-value = 55.74, R^2^ = 0.118) and in the 0–18 months period (linear: F = 4.72, R^2^ = 0.019; exponential: F = 7.73, R^2^ = 0.031). However, in the 18–36 months period, the results were non-significant. Please refer to Table 4 for more details.

**Table 4:** Model summary of the MD values according to no progress/progress (GPA) during the 3 years follow-up.

	Months	Equation	R^2^	F-value	*P*
MD No Progress	0–36	Linear	0.030	7.85	0.005*
		Exponential	0.051	11.76	0.001*
	0–18	Linear	N.A	N.A	0.42
		Exponential	N.A	N.A	0.15
	18–36	Linear	N.A	N.A	0.43
		Exponential	N.A	N.A	0.25
MD Progress	0–36	Linear	0.102	47.69	< 0.001*
		Exponential	0.118	55.74	< 0.001*
	0–18	Linear	0.019	4.72	0.031*
		Exponential	0.031	7.73	0.006*
	18–36	Linear	N.A	N.A	0.11
		Exponential	N.A	N.A	0.13

*MD* mean deviation, *GPA* guided progression analysis, *N.A.* non-applicable.

*Significant values.

Further, the study analyzed the whole cohort based on VFI values concerning GPA (no progress/progress). During the 3 years period of the study, no significant results were found among the non-progressors. The results remained non-significant when the group was divided into two (0–18, 18–36 months). However, significant results were observed for the linear and exponential functions in the 3 years and first periods (0–18 months) among the progressing patients (*p* ≤ 0.001/*p* = 0.002). In the total study time (3 years), the linear function was a better fit than the exponential function (linear: F = 48.47, R^2^ = 0.104; exponential: F = 44.03, R^2^ = 0.095). Conversely, in the first period (0–18 months), the exponential function showed a better fit than the linear function (linear: F = 10.14, R^2^ = 0.041; exponential: F = 11.76, R^2^ = 0.052). However, both equations showed non-significant results in the second period (18–36 months). Please refer to Table 5 for further details.

**Table 5:** Model summary of the VFI values according to no progress/progress (GPA) during the 3 years follow-up.

	Months	Equation	R^2^	F-value	*P*
VFI No Progress	0–36	Linear	N.A	N.A	0.16
		Exponential	N.A	N.A	0.22
	0–18	Linear	N.A	N.A	0.53
		Exponential	N.A	N.A	0.59
	18–36	Linear	N.A	N.A	0.77
		Exponential	N.A	N.A	0.79
VFI Progress	0–36	Linear	0.104	48.47	< 0.001*
		Exponential	0.095	44.03	< 0.001*
	0–18	Linear	0.041	10.14	0.002*
		Exponential	0.052	11.76	0.001*
	18–36	Linear	N.A	N.A	0.28
		Exponential	N.A	N.A	0.28

*VFI* visual field index, *GPA* guided progression analysis, *N.A.* non-applicable.

*Significant values.

## Discussion

In newly diagnosed exfoliation glaucoma patients, the visual field deterioration was not always linear, as per the results of the present study. Both functions, the linear and the exponential fitted well. There is a lack of evidence in the literature regarding the linearity of glaucoma progression in such patients. When examining the Mean Deviation (MD) values in the 36-month follow-up period, it was observed that the exponential function was a better fit than the linear function. However, when studying the Visual Field Index (VFI) values, the linear function was a better fit than the exponential function. The VFI values are transformed values that weigh the central parts of the visual fields more than the peripherals^14^. As the VFI values are already transformed, their linear nature may result from this transformation.

Between the glaucoma diagnosis and 18 months, the MD and VFI values changed exponentially. After this period, the visual fields’ deterioration slowed, resulting in a linear curve. This linear pattern is most likely due to the IOP-reducing effects that occur after 18 months, altering the natural course of the condition. Further analysis was conducted based on Glaucoma Progression Analysis (GPA), dividing patients into progressors and non-progressors. Interestingly, the exponential functions were the best fit for progressors due to the rapid deterioration of visual fields. However, only the MD values showed significance among non-progressors, while the VFI values among non-progressors were not significant. In summary, for both MD and VFI values, the curve changed exponentially between diagnosis and 18 months, then became linear due to the IOP-reducing effects. The best fit for progressors was an exponential function, while non-progressors showed significance only in the MD values.

The GPA analysis is the most commonly used clinical method by ophthalmologists in Sweden. It correlates well with point-wise linear regression (PLR) as described by De Moraes et.al. The present study found that patients with newly diagnosed exfoliation glaucoma (EXFG) have a high rate of visual field progression; around two-thirds of the glaucoma patients experienced progression within 3 years, based on the GPA analysis. These results align with another study that included 41 eyes of newly diagnosed primary open-angle glaucoma (POAG) patients. The authors found that 26.82% of the patients progressed according to the GPA over 2 years of follow-up. The present study confirmed previous results that have identified exfoliation glaucoma as an aggressive form of glaucoma. However, it is important to note that the patients included in this study were newly diagnosed, and disease progression may have slowed down later on^15–18^.

Numerous studies have previously described the various risk factors associated with the development and progression of glaucoma^6,19,20^. However, developing predictive models for glaucoma progression has proven challenging. This is primarily due to the significant variability among patients and the unique progression of different types of glaucoma. During the 3 year follow-up period of the present study, there was a significant variability in the MD and VFI values, as evident from the data spread in Figs. 4 and 5. Another problem in building predictive models lies in the differences in data collection methods across various studies. Moreover, the generalizability of predictive models is based on internal and external validity, which is difficult to achieve in glaucoma progression studies due to differences in patient populations. However, the present study provides crucial insights into glaucoma progression that can aid in the development of predictive models in the future.

Patients with advanced visual field progression had higher IOP values than those without progress. This finding aligns with previous studies that identified IOP as a well-known risk factor for glaucoma progression^19^. Additionally, age was another factor that differed between the groups, with older patients exhibiting more advanced progression than younger patients. This finding is also consistent with previous studies^6^. During the 3 years follow-up period, patients with advanced progression had more deteriorated visual fields at inclusion than non-progressors. This study found that progressors and non-progressors had significantly different MD and VFI values at the start. This aligns with previous research, indicating that patients with more damaged visual fields experience greater progression^21^. The majority of patients (69%) presented with unilateral exfoliation glaucoma at inclusion. No patient conversed from unilateral till bilateral during the study follow-up. Unilateral presentation of EXFG found in the present study is consistent with previous studies^22^.

There were some limitations to the present study. Glaucoma progression was measured using visual fields, which remains the gold standard for evaluating disease progression^8^. No anatomical measurements of the optic nerve were performed (like Optical Coherence Tomography). The patients included in the study were all diagnosed with exfoliation glaucoma; therefore, the findings may not apply to other types of glaucoma. Additionally, all patients were born in Sweden, and genetic factors may have been involved, so the study results may not apply to other populations. It is important to note that the study did not involve patients with advanced glaucoma damage, as it would have been challenging to follow up with them. As a result, our findings only apply to patients with early or moderate glaucoma. Additionally, it is necessary to consider selection bias, as patients who did not cooperate in the visual field examinations were not included. Nevertheless, the number of patients excluded due to non-cooperation was minimal.

In conclusion, it has been observed that patients newly diagnosed with exfoliation glaucoma experience an exponential progression in the period between diagnosis and 18 months. However, the effects of reducing intraocular pressure (IOP) through treatment can transform this curve into a linear function. Previous studies have assumed that glaucoma progression follows a linear pattern. The present study showed that exponential progression might be considered at the beginning of the disease. Information coming from the present study can be valuable for establishing predictive models for exfoliation glaucoma progression in the future.

## Data Availability

The datasets generated during and/or analysed during the current study are available from the corresponding author on reasonable request.
